# Annotations

**Published:** 1897-10-30

**Authors:** 


### Annotations.

### The Carriers of Diphtheria.

The length of time during which the bacillus of diptheria may continue in the throat after an attack of the disease is a matter of the greatest interest, for on it depends to a large extent the possibility of arresting the progress of diphtheria by means of isolation. But an even more important difficulty in tracing the origin of infection and in preventing its importation arises from the fact that the bacillus may exist in a state of virulence in the throats of apparently healthy people. A person, for example, who happens to be immune to the disease, at any rate for the time, is exposed to infection. The microbes lodge and develop, and remain virulent. No disease, however, is produced, and nothing happens to attract attention to the fact that this person is a carrier of infection. It is to be feared that it is practically impossible to eliminate this source of disease. Of course, the chief means by which immunity is produced is a comparatively recent attack of the malady itself. In such cases when the attack is over, but the microbes may remain, and in consequence of the patient's acquired immunity he may feel no inconvenience. The most striking instance of this condition is a French case, in which the bacillus was still to be found at the end of fifteen months. Another case is reported in which Dr. Hewlett found the bacillus at intervals during twenty-two weeks; and its persistence for three or four weeks after apparent convalescence is by no means uncommon. Now it is easy to say that such cases must not be allowed to mix with other people until their throats have been shown to be free from infection, but a much more serious difficulty looms behind. These immune individuals, if exposed to infection again, may catch it afresh, and carry it about with them without showing any signs. A case is reported by Dr. Foulerton, of the British Institute of Preventive Medicine, in which this sequence of events occurred, and a consideration of the facts of the case makes it quite obvious how enormously difficult it must be to obtain effective control over an infection which is liable to take root and be distributed in such a manner.

### Curiosities of Filtration.

In Sir E. Frankland's Annual Report on Metropolitan Water just published by the Local Government Board some very curious details may be found in regard to the results of the filtration to which London water is subjected, all of which tend to support the statements recently made by The Hospital as to the extreme variability of the filtered product. Take, for example, the West Middlesex, which month after month supplies its customers with water of a high degree of purity, containing on one occasion only four microbes per cubic centimetre, and on another appearing to be absolutely sterile. Of what advantage, however, is this if on another occasion the number mounts up to 120, and on still another to 576 microbes per cubic centimetre? Something happened in the month of June to nearly all the filters. "Of the five companies drawing from the Thames, all except the Southwark were smitten with this microbial epidemic in June, and even the Southwark had got it on the 2nd of the following month. Of the two companies drawing from the Lee, the New River alone escaped." So serious was the condition that from the tables given to show the reduction of micro-organisms by filtration alone we find that in one case 66·3 per cent. of the microbes passed the filters. Lest, however, we should be tempted to cast ourselves upon Providence in these matters, and think that this "microbial epidemic" was some widespread fatality that no company could escape from, it is worth while to look further into the matter, when we find that where separate filter-beds were separately examined, as we have maintained ought always to be done, a very great difference was demonstrated in their activity. While one of the Grand Junction filters was passing 16 and another 56 microbes per c.c., another was passing 1,080! What this has to do with the construction of the filters and what can be done to improve them is another matter; but Sir E. Frankland seems to be on the right track when he draws attention to "the enormous advantage of fine sand in securing efficient filtration." Some companies go to the trouble of using much finer sand than others with apparently good results. "Thus: 1·8 foot of the fine sand of the New River Company and 2·75 feet of that of the West Middlesex Company are respectively more than twice as efficient as 4 feet of the coarser material used by the Chelsea Company."

### Shot-Gun Quarantine.

The southern portion of the United States appears to have been suffering from an epidemic of "shot-gun quarantine," as well as from one of yellow fever. Not only has each State made its own rules and regulations for the control of the yellow fever, but each town and city has tried to exclude from its precincts all whom it chose to consider liable to be tainted with infection. The shot-gun has been called into play as a means of enforcing the cordon, panic-stricken citizens have resorted to mob rule in "holding up" railway trains, interfering with traffic, arresting Government experts, hindering food supplies, and attacking temporary hospitals. Only last week we heard of a mob attacking and attempting to burn a school-house rather than allow it to be used as a temporary hospital. The position is becoming intolerable, and we do not wonder that a demand is arising for some national system, instead of the present local measures for dealing with outbreaks of infectious disease. The demand, however, takes an unfortunate form, for the cry is raised for a "national quarantine," a term which suggests a much discredited mode of sanitary defence—really only an attempt to do on a larger scale what the deluded citizens of outlying towns are now striving to effect, in their own crude manner by aid of shot-guns. It is probable, however, that when the American papers ask for a national system of quarantine, they use the term in a much larger sense than that in which it is usually employed, and really mean a system of inspection of travellers and isolation of the sick such as has been found effective in dealing with cholera and suchlike diseases in other parts of the world.

